# Systemic Inflammatory Effects of Traumatic Brain Injury, Femur Fracture, and Shock: An Experimental Murine Polytrauma Model

**DOI:** 10.1155/2012/136020

**Published:** 2012-03-04

**Authors:** C. Probst, M. J. Mirzayan, P. Mommsen, C. Zeckey, T. Tegeder, L. Geerken, M. Maegele, A. Samii, M. van Griensven

**Affiliations:** ^1^Department of Trauma and Orthopaedic Surgery, Cologne Merheim Medical Center, Faculty of Health-School of Medicine, Witten/Herdecke University, Ostmerheimer Straße 200, 51109 Cologne, Germany; ^2^Department of Trauma, Neurosurgery, Hannover Medical School, 30623 Hannover, Germany; ^3^Department of Traumatology, Hannover Medical School, 30623 Hannover, Germany; ^4^Department of Anaesthesiology, Nordstadt Hospital Hannover, 30167 Hannover, Germany; ^5^International Neuroscience Institute Hannover, Department of Neurosurgery, 30625 Hannover, Germany; ^6^Institute of Experimental Traumatology, University of Technology-Munich, 81675 München, Germany

## Abstract

*Objective*. Despite broad research in neurotrauma and shock, little is known on systemic inflammatory effects of the clinically most relevant combined polytrauma. Experimental investigation in an animal model may provide relevant insight for therapeutic strategies. We describe the effects of a combined injury with respect to lymphocyte population and cytokine activation. *Methods*. 45 male C57BL/6J mice (mean weight 27 g) were anesthetized with ketamine/xylazine. Animals were subjected to a weight drop closed traumatic brain injury (WD-TBI), a femoral fracture and hemorrhagic shock (FX-SH). Animals were subdivided into WD-TBI, FX-SH and combined trauma (CO-TX) groups. Subjects were sacrificed at 96 h. Blood was analysed for cytokines and by flow cytometry for lymphocyte populations. *Results*. Mortality was 8%, 13% and 47% for FX-SH, WD-TBI and CO-TX groups (*P* < 0.05). TNF*α* (11/13/139 for FX-SH/WD-TBI/CO-TX; *P* < 0.05), CCL2 (78/96/227; *P* < 0.05) and IL-6 (16/48/281; *P* = 0.05) showed significant increases in the CO-TX group. Lymphocyte populations results for FX-SH, WD-TBI and CO-TX were: CD-4 (31/21/22; *P* = n.s.), CD-8 (7/28/34, *P* < 0.05), CD-4-CD-8 (11/12/18; *P* = n.s.), CD-56 (36/7/8; *P* < 0.05). *Conclusion*. This study shows that a combination of closed TBI and femur-fracture/ shock results in an increase of the humoral inflammation. More attention to combined injury models in inflammation research is indicated.

## 1. Introduction

Trauma and especially multiple trauma including traumatic brain injury (TBI) are the most common killers in children and adults before age 50 around the world [1–3]. Mortality of multiple trauma including TBI following traffic accidents ranges between 18% and 25% [1, 4]. But not only the first hours and days are critical; for years, more and more multiple trauma patients have been surviving the initial accident and acute care period but only to develop systemic inflammatory response syndrome (SIRS), sepsis, or multiple organ dysfunction syndrome (MODS) in the course of the few weeks of critical care [2, 5, 6].

For 5000 multiple trauma patients in Germany, the German Trauma Registry showed an in-hospital mortality of 12% in 2010. In the same report, critical care complications such as SIRS, sepsis, and MODS are reported for 15% of patients. Other groups report similar rates of SIRS, sepsis, and MODS [2, 6, 7]. It is generally known that these complications again deteriorate the prognosis and outcome of the initially surviving trauma patient and increase late mortality to up to 50% if manifest MODS is developed [2, 7, 8].

To understand pathophysiological processes leading to multiple organ failure, SIRS, and sepsis and taking place during this devastating illness, profound pathophysiological knowledge on the effects of the initial trauma (“first hit”) is essential to minimize additional harm by the following surgical or critical care treatment (potential “second hit”) [9–11].

Therefore, numerous neurotrauma as well as shock research studies with many different animal and clinical models showed inflammatory changes after trauma [12–14]. Only recently, Semple et al. reported on the important role of CCL2 in C57Bl/J6 mice, peaking at 4 to 12 h following closed head injury and leading to improved functional outcome and lesion size reduction as well as less secondary brain damage [15]. Another group showed that the substance Minozac may attenuate the effect of proinflammatory cytokines after closed TBI in mice. In the same study, the authors discussed if Minozac may also increase long-term neurofunction after TBI [16]. On the other hand, femur fracture and shock in mice are known to trigger an inflammatory response for years, and recently several groups discussed therapeutic strategies to modulate this inflammatory response [12, 14].

However, little attention has been paid to the immune reaction following combined trauma and the interaction of the brain and the other organs of the body. This is even more important, since the injury pattern of closed TBI, lower extremity fracture, and shock is very common in multiply injured patients treated in trauma centers around the world. Therefore, we investigated the humoral and cellular inflammatory changes of the combined TBI, shock, and femur fracture in a polytrauma model of the mouse.

## 2. Material and Methods

### 2.1. Animal Care

The study was approved by the Animal Research Committee of the Hannover Medical School (Medizinische Hochschule Hannover, MHH) and the county government of Lower Saxony (Bezirksregierung Niedersachsen), no. 03/672.

45 male C57BL/J6 mice aged 8–10 weeks with a mean weight of 27.4 g (±3.7 g) were used for the study. The animals were bred and raised in the central animal facility of our institution. All animals were handled at room temperature for 7 days before treatment. Throughout the study period, pelleted mouse feed (Altromin 1324) and water were available ad libitum. The lighting was maintained on a 12-hour cycle. Room temperature was maintained at a constant 20° ± 2°C.

### 2.2. Anesthesia

Analgetic therapy during the whole time of the study was provided by Metamizol 25 mg/1000 mL (Novaminsulfon ratiopharm, Ratiopharm GmbH, Ulm) in the drinking water of the animals ad libitum.

All surgical and trauma procedures were performed at a level of deep surgical anesthesia during maintained spontaneous breathing. For anesthesia, subcutaneous injection of Ketamine (Ketanest, Parke-Davis GmbH, Karlsruhe, Germany) with a dose of 100 mg/kg body weight and 2% Xylazine (Rompun, Bayer Vital GmbH, Leverkusen, Germany) with a dose of 16 mg/kg body weight were used. The eyes of the animals were covered by Dexpanthenol (Bepanthen, Hoffmann-La Roche AG, Grenzach-Wyhlen, Switzerland). The trauma and perioperative care was performed under an infrared heating lamp. Thereby body temperature of 36°C could be maintained.

### 2.3. Weight Drop Traumatic Brain Injury (WD-TBI)

30 of the animals underwent a standard WD-TBI following a model published previously by Shapira et al. [17] with modification according to Chen et al. [18]. Under a surgical level of anesthesia, the animal was positioned in a stereotaxic frame, the head was shaved, and the exposed scalp disinfected by Softasept N (Braun, Melsungen, Germany). A midline incision was performed, and the skull was exposed. Next, the 3 mm diameter probe was positioned on the planned impact point at the left of the sagittal suture and caudad to the coronal suture. The trauma was now performed with a mean velocity of 3 m/s by a software-guided process (“Labview,” National Instruments, Austin, TX, USA).

Animals were taken out from the stereotaxic frame while still anesthetized. The wound was irrigated and closed by 4.0 prolene sutures. (Ethicon, Norderstedt, Germany). Afterwards, the animals remained under continuous observation and warming until full recovery was reached.

### 2.4. Femur Fracture

Femur fracture was applied by a blunt guillotine device according to Van Griensven et al. [19], We used a weight of 400 g falling out of a height of 160 mm on the fixed femur. Success of the fracture mechanism was controlled clinically (crepitation and instability), while the animal was still in anesthesia. If both of the clinical signs were absent, the trauma was repeated. The repeated procedure was required in one animal only. The guillotine procedure resulted in an A-type femur fracture combined with a moderate soft tissue injury. After completion of the injury, we performed a closed reduction and splinting of the fracture by a piece of wood in proper length that was tied to the fractured extremity proximally and distally of the fracture.

### 2.5. Shock

After both traumata were set, we draw 60% of the blood volume of the respective mouse via an orbital puncture with a heparinized capillary. Volume was restored after 1 hour of shock with Ringer's solution four times the extracted blood volume into the tail vein. This resulted in an individual resuscitation procedure. Only after this resuscitation was completed and the animal showed 36°C of body temperature, anesthesia was terminated and the animal awoke [19].

### 2.6. Analysis of the Cytokines and Lymphocytes

Subjects were sacrificed 96 hours after trauma by exsanguination in deep surgical anaesthesia, while blood samples for cytokines were extracted. As well the spleen was harvested, and a suspension was produced. This suspension was used for lymphocyte analysis as described earlier [19]. Lymphocytes were analyzed using standard flow cytometry (FACS analysis) for CD-4, CD-8, CD-4-CD-8, and CD-56 cells (FACS Calibur, BD Biosciences, Heidelberg, Germany). The procedures have been described earlier [19].

Serum cytokines (tumor necrosis factor *α*, TNF*α*; chemokine CC2 ligand, CCL2; interleukins 6 and 12, IL-6 and IL-12) were analyzed by cytometric bead array technique (BDTM Cytometric Bead Array Mouse Inflammation Kit, BD Biosciences, Pharmingen, Germany) and FACS as described earlier [19].

### 2.7. Statistics

Analysis of continuous parameters was by ANOVA and a post hoc Tukey test. Mortality ratios were analyzed by chi-squared test. Results are reported as mean ± standard error of the mean (SEM). For statistical analysis, SPSS 14.0 software was used. Statistical significance was set at *P* < 0.05.

## 3. Results

### 3.1. Mortality

**Figure 1:**
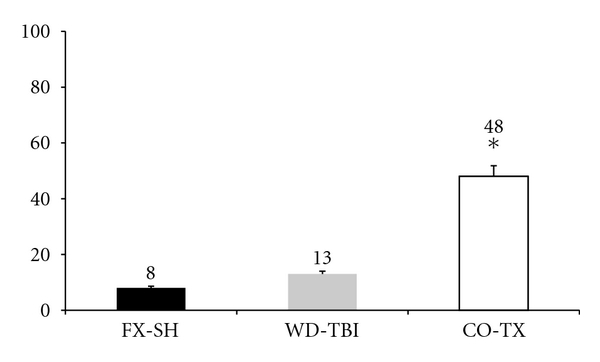
Mortality rates (%) of the 3 trauma subgroups. FX-SH: femur fracture and shock; WD-TBI: weight drop traumatic brain injury; CO-TX: combined injuries. (∗)*P* = 0.0263. The CO-TX group showed a significantly higher mortality.

The polytraumatized animals showed a significantly higher mortality than the animals of both singular trauma groups (Figure 1). There was no immediate death. Deaths occurred between 24 and 72 hours after trauma.

### 3.2. Humoral Inflammatory Markers

**Figure 2:**
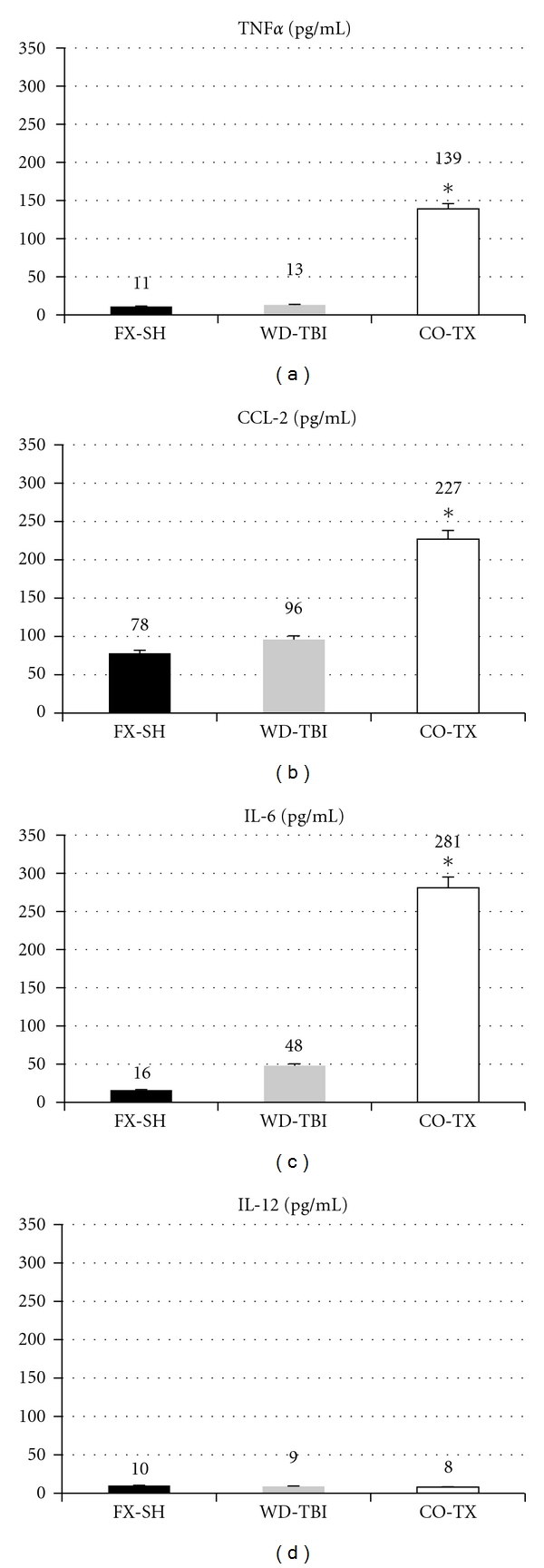
Cytokine levels (pg/mL) of the three subgroups. FX-SH: femur fracture and shock; WD-TBI: weight drop traumatic brain injury; CO-TX: combined injuries. (∗)*P* < 0.05.

TNF*α*, CCL2, and IL-6 showed significantly higher concentrations at 96 h after trauma in the CO-TX group, while the single-trauma groups seem to raise their cytokine levels clearly less and without significant differences between groups WD-TBI and FX-SH (Figure 2). IL-12 showed no difference in plasma concentration between the groups at 96 h after trauma.

### 3.3. Lymphocyte Populations

**Figure 3:**
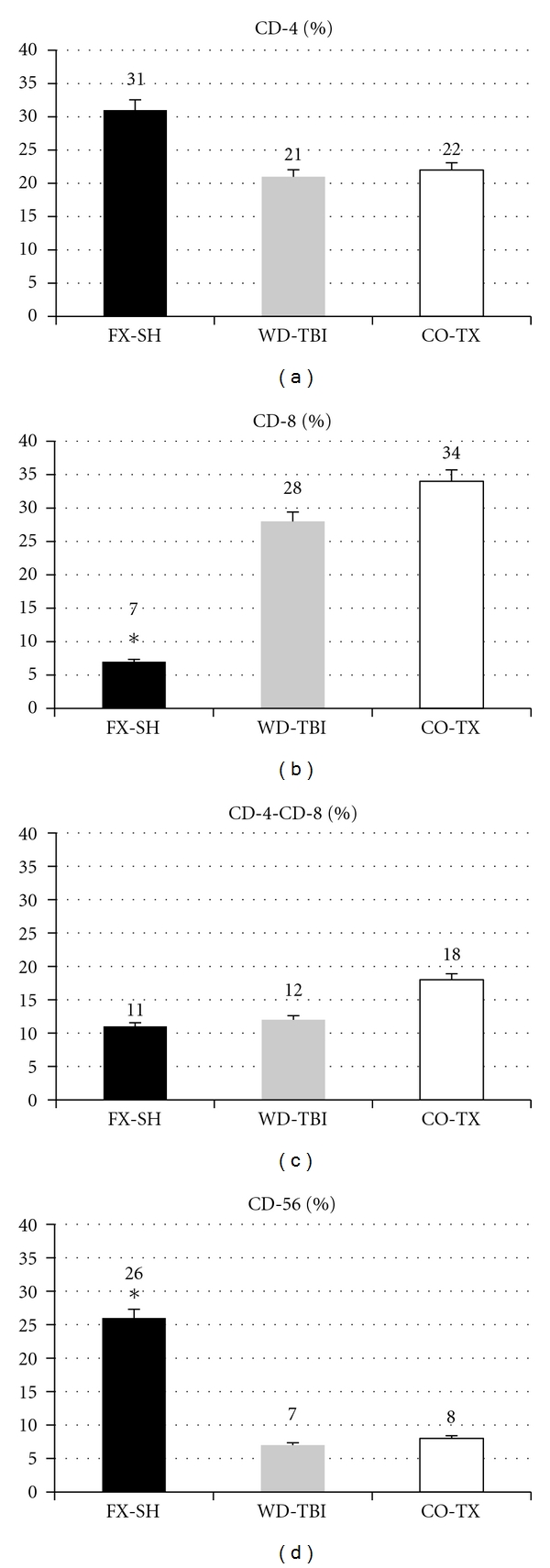
Lymphocyte subpopulations (%). FX-SH: femur fracture and shock; WD-TBI: weight drop traumatic brain injury; CO-TX: combined injuries.

The CD-8 ratio was significantly lower, and the CD-56 ratio was significantly higher in the FX-SH mice than in each of the other groups. CD-4 and CD-4-CD-8 ratios showed no difference between groups (Figure 3).

## 4. Discussion

While the intracranial effects of traumatic brain injury have been a subject of multiple studies, the aim of this study was to investigate the interaction of the brain injury and systemic shock after lower extremity trauma in regard to changes in the inflammatory response.

Our main results were:

1. increased levels of TNF*α*, CCL2, and IL-6 in the CO-TX group,
2. lower rates of CD-8 and higher rates of CD-56 cells in the FX-SH group,
3. an increased mortality in the combined trauma animal group.

However, some weaknesses of our study have to be kept in mind. As always in animal studies, the findings cannot completely be extrapolated to human pathophysiology and clinical treatment strategies. Nonetheless, mouse models are very common due to their availability, ease of handling, and practicability. Anyhow, a correlation with similar large animal models and additional studies in humans are required to complete our findings and value the clinical relevance. Furthermore, the injury pattern may seem artificial in its constellation since one huge challenge of human polytrauma studies is the diversity of injury patters requiring complex inclusion and exclusion procedures to gain comparable populations in terms of injury severity and injury pattern. However, in our point of view, this strengthens our study: the injury pattern and severity of our individuals is highly standardized, and the model and both its components are robust and reproducible. So comparability of our injuries and populations is granted, even more so with regard to the complex hypothesis of a combined trauma being more than only the sum of its parts. This holds even true for standard parameters such as weight, size, sex, and age, that usually also affect human studies of polytrauma patients.

Our first finding supports any injury severity-related changes in inflammatory response reported from humans [2, 20–22]. Our CO-TX group simply suffered more severe injuries than the FX-SH and WD-TBI groups. Looking at each monotrauma, our plasma cytokine levels are comparable to those reported earlier in literature [12, 14]. However, the levels of the proinflammatory cytokines are elevated not only by factor 2 but by factors 3 to 10, respectively, even as late as 96 hours after trauma. Other groups found similar results: TNF*α* was associated with injury severity earlier. Likewise, in a murine trauma-haemorrhage model, other authors reported TNF*α* elevations [23, 24]. The same holds true for TBI in humans [25, 26] and clinically relevant models of TBI [27]. While these groups reported elevated TNF*α* levels 1 to 4 h after trauma, later publications also found TNF*α* effects on neuronal recovery and prevention of secondary brain damage [28]. One group reported elevated plasma TNF*α* levels after TBI. So, while the importance of TNF*α* after peripheral or brain injury remains rather clear and reciprocal effects were described to some extent, we found no earlier report on the extent of TNF*α* plasma level rise after combined TBI and trauma haemorrhage. Concerning IL-6, Maegele et al. reported a 3-4 fold increase in circulating plasma levels after combined fracture and fluid percussion TBI in rats [29]. In humans, IL-6 was classified as the most important secondary cytokine in trauma [30], not only because of its multitude of mainly proinflammatory effects and its association to the severity of trauma, but also because of its prognostic relevance differing survivors and nonsurvivors. So, highest IL-6 levels in the most severe trauma group and the highest mortality are in line with earlier results in literature. However, the magnitude of the increase was rarely described. Similarly, CCL-2 is increased in the brain after TBI and leads to increased migration of intravascular leukocytes across a damaged blood-brain barrier [31, 32]. As well CCL-2 was found to play a key role in the development of organ dysfunction after trauma by activating monocytes/macrophages to migrate from the intravascular space into peripheral tissues. While Thibeault et al. showed no association between IL-6 and CCL-2 after trauma, they could show such an association between TNF*α* and CCL-2 [33]. So, while being in line with recent literature, our findings also remain conclusive within themselves with respect to the inflammatory cascades described earlier. With respect to proinflammatory IL-12, our nonsignificantly low serum levels are backed up by recent literature. In TBI, one author described differences in IL-12 after 24 h but not later [30]. In human multiple trauma patients suffering from chest trauma, IL-12 was reported to inversely correlate with mortality [30]. Furthermore, IL-12 seems to exert some major effects via CD56+-(NK-) cells [30]. However, depressed IL-12 production in trauma patients was associated with a shift towards a CD4+(TH2) type pattern of adverse clinical outcome [30].

While numerous reports focus on intravascular lymphocyte populations and function after trauma, the populations crucial for MODS to develop are those in peripheral tissues [30]. The significant decrease of the CD-8+ ratio in the splenic suspension of our FX-SH group is supported by the earlier finding of a decreased ratio of CD-8+ lymphocytes in peripheral tissues after trauma hemorrhage compared to controls [34]. However, we found no report on lymphocyte populations in peripheral tissues after traumatic brain injury. Yet, the WD-TBI group might be comparable to controls for peripheral trauma and hemorrhage (our FX-SH group) and therefore shows higher CD-8+ ratios. Still, this hypothesis does not explain the CD-8+ ratio of the CO-TX group. Restoration of the low CD-8+ ratio after FX-SH by WD-TBI might be speculated on but so far no theory on responsible mechanisms is known. Since this result was rather surprising for our group, detailed evaluation of key processes for lymphocyte populations after combined neuro- and peripheral trauma and hemorrhage must be left for future experiments. CD-56-(NK-) cells were found in a significantly higher concentration in the splenic suspension of our FX-SH group compared to our WD-TBI and CO-TX groups. This also is supported by earlier results as in CD-8+ cells [34]. While the same reasons as above for the physiologic CD-56 ratio in our WD-TBI group may be discussed, in the CO-TX group one more aspect needs consideration: raised TNF*α* levels in a murine sepsis model were associated with an increased ratio of NK cells [35, 36]. However, for CD-56+ cells as well as for CD-8+ cells, the detailed mechanisms and reasons underlying the differences found in our study cannot readily be explained. Further experiments should focus on lymphocyte populations in peripheral tissues after TBI and combined TBI and trauma hemorrhage.

Mortality may just reflect the inflammatory changes and/or trauma load [1, 12]. In humans, injury severity clearly is associated with mortality. Likewise, injury severity was associated with an increased inflammatory response as measured by increased IL-6 levels. So was mortality, an increased IL-6 level in multiply injured patients was associated with increased mortality. IL-6 even was suggested to be used for prognosis in severe trauma [2, 30]. In murine models, other groups support the findings in humans and of our study [12, 14, 37].

## 5. Conclusion

Our animal model of combined extremity fracture, shock, and closed traumatic brain injury clearly showed that the inflammatory response of the combined trauma is severalfold elevated as compared to the respective monotrauma in terms of humoral parameters. The almost physiological findings for peripheral tissue lymphocyte populations in the isolated TBI and in the combined trauma group as compared to the significantly elevated ratios of the trauma-hemorrhage group are not readily explained and need further evaluation. Mortality in our combined trauma model is clearly associated with trauma load and inflammation and such parallels the findings in multiple trauma patients. This shows the value of our model for future investigations.
